# Assessment of psychological defense mechanisms in women with somatoform disorder using Thematic Apperception Test-Based Measure

**DOI:** 10.1192/j.eurpsy.2022.984

**Published:** 2022-09-01

**Authors:** N. Lebedeva, Y. Chebakova, A. Parshukov

**Affiliations:** 1 Moscow Metropolitan Governance University, Diagnostics Department, Moscow, Russian Federation; 2 Moscow Institute of Psychoanalysis, Research Department, Moscow, Russian Federation

**Keywords:** thematic apperception test, somatoform disorder

## Abstract

**Introduction:**

Maladaptive defense mechanisms can play a role in maintaining the inadequate social and psychological adaptation of patients.

**Objectives:**

This study aims to establish if denial is one of the central psychological defense mechanisms in patients with somatoform disorder.

**Methods:**

10 female patients at Moscow Clinical hospital №33 with somatoform disorder and panic attacks (aged 20 to 43) and 20 female participants of the control group (aged 19 to 35) were presented with 10 pictures of the Thematic Apperception Test. Pictures were previously annotated into 4 groups: neutral stimuli (2, 6GF), provoking self-blame / depression ideation stimuli (3GF,14, 15, 17GF), provoking aggression ideation stimuli (8ВМ, 18 GF, 9GF), provoking aggression/self-blame ideation stimuli (13 MF). We conducted content analyses of stories. Mann-Whitney U-test was used.

**Results:**

Table 1 presents analyses categories, examples of stories, and group differences.

**Table 1:** 

Category	Example	Patients,% of stories	Control group,% of stories	Mann-Whitney U-test
Denial of interpersonal/internal conflict	«It is a beautiful day. The girl is enjoying the sunlight. Her life is going well» (17GF).	90%	47%*	р<0,01
Denial of aggressive ideation	«She won`t smother her, she just wants to scare her a little» (18 GF).	70%	30%	р<0,05
Denial of depressive /self-blame ideation	«Is she dead or not? I think, no. They were having sex and now they are sleepy» (13MF).			

* Several patients told more than 1 story to a picture.

**Conclusions:**

Patients with the somatoform disorder tended to use descriptions without interpersonal or internal conflicts and/or to deny any characters‘ negative intentions or the negative consequences of their actions.

**Disclosure:**

No significant relationships.

